# Water/Alcohol Soluble Thickness-Insensitive Hyperbranched Perylene Diimide Electron Transport Layer Improving the Efficiency of Organic Solar Cells

**DOI:** 10.3390/polym11040655

**Published:** 2019-04-10

**Authors:** Dan Zhou, Fei Yang, Yuancheng Qin, Rong Zhong, Haitao Xu, Yongfen Tong, Yubao Zhang, Qin Zhang, Mingjun Li, Yu Xie

**Affiliations:** 1 Key Laboratory of Jiangxi Province for Persistent Pollutants, Control and Resources Recycle, Nanchang Hangkong University, 696 Fenghe South Avenue, Nanchang 330063, China; 15770938986@163.com (F.Y.); qinyuancheng@hotmail.com (Y.Q.); zhongr@nchu.edu.cn (R.Z.); 40019@nchu.edu.cn (Y.T.); limingjun@nchu.edu.cn (M.L.); 2 College of Materials Science and Engineering, Nanchang Hangkong University, 696 Fenghe Avenue, Nanchang 330063, China; haitaoxu@nchu.edu.cn; 3 School of Measuring and Optical Engineering, Nanchang Hangkong University, Nanchang 330063, China; 70637@nchu.edu.cn (Y.Z.); 70433@nchu.edu.cn (Q.Z.)

**Keywords:** thickness-insensitive, electron transport layer, perylene diimides

## Abstract

The electron transport layer (ETL) is very crucial for enhancing the device performance of polymer solar cells (PSCs). Meanwhile, thickness-insensitive and environment-friendly water/alcohol soluble processing are two essential requirements for large-scale roll-to-roll commercial application. Based on this, we designed and synthesized two new n-type ETLs with tetraethylene pentamine or butyl sulfonate sodium substituted tetraethylene pentamine as the branched side chains and high electron affinities perylene diimide (PDI) as the central core, named as **PDIPN** and **PDIPNSO_3_Na**. Encouragingly, both **PDIPN** and **PDIPNSO_3_Na** can effectively reduce the interfacial barrier and improve the interfacial contact. In addition, both of them can exhibit strong n-type self-doping effects, especially the **PDIPN** with higher density of negative charge. Consequently, compared to bare ITO, the PCE of the devices with ITO/**PDIPN** and ITO/**PDIPNSO_3_Na** ETLs has increased to 3–4 times. Our research results indicate that n-type self-doping PDI-based ETL **PDIPN** and **PDIPNSO_3_Na** could be promising candidates for ETL in environment-friendly water/alcohol soluble processing large-scale PSCs.

## 1. Introduction

Polymer solar cells (PSCs) have attracted increasing attention due to their lightweight, flexibility, and easy large-area solution processability [1,2,3,4,5,6,7,8,9,10,11]. With the rapid progress in novel donor and acceptor material design and interfacial engineering, the power conversion efficiency (PCE) of the PSCs have been boosted over 14% [12,13]. Currently, most research focuses on the design of novel active layer materials for improving the power conversion efficiency (PCE) of the PSCs [14,15,16,17,18,19]. Interfacial modulating is also very crucial for enhancing the PCE of the device [20,21,22,23,24].

The mismatched energy lever between the active layer and electrode can cause the Schottky barrier, thus inhibiting the charge transfer and injection [25]. Suitable interfacial modification can decrease the interfacial barrier and increase the selectivity of charge, thus inhibiting the charge recombination and increasing the efficiency of charge injection [26,27]. Hence, inserting an interlayer between active layer and cathode electrode should be an effective strategy to increase the performance of the device. To date, metal oxide semiconductor (TiO_x_, ZnO, MoO_x_), electrolytes and fullerenes derivates etc have been used as ETLs to modulate interfacial contact and reduce interfacial barriers [28,29,30,31,32,33,34]. However, inorganic metal oxide ETL usually shows incompatible with the organic light harvest. Moreover, most of inorganic metal oxide ETLs need to be treated with a high temperature, which cannot be satisfied with the demand of the solution-process large-scale commercial application.

In consequence, to explore environment-friendly water/alcohol soluble processing organic ETL with strong interfacial control capability is extremely urgent. It would be better if the ETL is thickness-insensitive and can be efficient in different interlayer thickness. Perylene diimide (PDI) is a famous n-type material, which has many strong points, such as good electron affinities, high conductivities and facile modification by simple substitution reaction [35,36,37,38]. Moreover, the high conductivity of the PDI originated from self-organized p-stacks in solid film can endow the PDT-based ETL with thickness-insensitive superiority, which can conquer the thickness restriction (<10 nm) of the reported organic ETL. Thus, PDT-based ETL may have the potential to realize large-area manufacturing techniques owing to its tolerance of certain thickness variation.

**Scheme 1:**
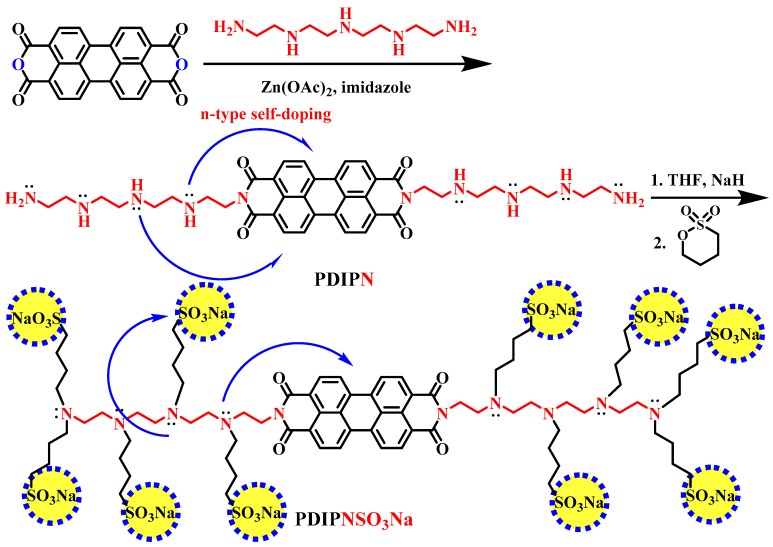
Synthetic routes of **PDIPN** and **PDIPNSO_3_Na**.

N-type self-doping phenomena of the ETLs can enhance the electron mobility and form additional big interfacial dipoles on the interface of ITO and active layer [39]. Combining the PDI and n-type self-doping at molecular level can endow the material with the advantages of both materials. Tetraethylene pentamine contains enough polar groups and unpaired electrons of the N atoms. Inspired by this, we designed and synthesized two new n-type ETLs **PDIPN** and **PDIPNSO_3_Na** (Scheme 1) with perylene diimide (PDI) as central core and tetraethylene pentamine or butyl sulfonate sodium substituted tetraethylene pentamine as the branched side chains. Intriguingly, both n-type ETLs **PDIPN** and **PDIPNSO_3_Na** can form favorable interfacial dipole due to the polar groups and n-type self-doping effect. In sharp comparison to the device without ETL, the PCEs of the inverted device based on ITO/**PDIPN** and ITO/**PDIPNSO_3_Na** have been increased over two times. Furthermore, both **PDIPN** and **PDIPNSO_3_Na** can obtain an acceptable efficiency among a wide range of ETL thickness. As a result, environment-friendly water/alcohol soluble thickness-insensitive **PDIPN** and **PDIPNSO_3_Na** should be a promising ETL for large-scale commercial application.

## 2. Synthesis and Characterization

**Figure 1:**
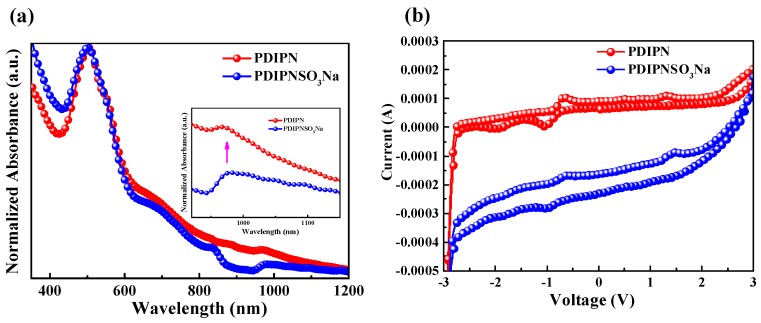
(**a**) UV–Vis absorption and (**b**) cyclic voltammograms (CV) spectra of **PDIPN** and **PDIPNSO_3_Na** films.

The detailed synthetic procedures of the hyperbranched perylene diimides ETLs **PDIPN** and **PDIPNSO_3_Na** are provided in Scheme 1, and the synthetic route is depicted in the Supporting Information. The **PDIPN** is synthesized by one-step substitution reaction between 3,4,9,10-perylenetetracarboxylic diimides and tetraethylene pentamine with a catalytic amount of zinc acetate (127 mg). The **PDIPNSO_3_Na** is prepared by **PDIPN** with NaH and 1,4-butanesultone via simple substitution reaction. Due to the existence of the polar group, the **PDIPN** and **PDIPNSO_3_Na** ETL can realize non-polluted water/alcohol processing. The structures of **PDIPN** and **PDIPNSO_3_Na** are verified by ^1^H nuclear magnetic resonance spectra (^1^H NMR) (Figure S1–S2) and UV–Vis absorption (Figure 1).

To calculate the optical bandgap (E_g_^opt^) and electrochemical bandgap (E_g_^ec^) of the ETLs **PDIPN** and **PDIPNSO_3_Na**, the UV−Vis and cyclic voltammetry (CV) are investigated, as shown in Figure 1. From Figure 1a, we can obviously see that the absorption band onset (λ_onset_) of **PDIPN** and **PDIPNSO_3_Na** are at 614 nm and 620 nm, an optical bandgap (E_g_^opt^) can be calculated to be 2.02 eV for **PDIPN** and 2.00 eV for **PDIPNSO_3_Na**. Meanwhile, a broad absorption peak can be easily observed at 800 nm and extended to 1100 nm both in **PDIPN** and **PDIPNSO_3_Na** film, which should be ascribed to the absorption of polarons [40,41,42]. These results suggest that maybe there are n-type self-doping effects exist in **PDIPN** and **PDIPNSO_3_Na** owing to the existence of unpaired electron in N atoms [43]. The unpaired electron of the N atoms can transfer to the perylene diimidess nucleus, thus giving rise to the formation of n-type self-doping behavior. Furthermore, compared to **PDIPNSO_3_Na**, **PDIPN** film shows stronger absorption band of polarons, indicating that the number of free electron of **PDIPN** is much more than that of **PDIPNSO_3_Na**. Because one mole **PDIPNSO_3_Na** has ten moles electron-withdrawing sodium sulfonate, the density of free electrons of **PDIPNSO_3_Na** is lower than that of **PDIPN**. The n-type self-doping can form additional interfacial dipole, which is beneficial to reduce the interface barrier and enhance the electron injection efficiency, thus increasing the PCE of the device. The electrochemical energy levels are measured by cyclic voltammetry in an anhydrous nitrogen-saturated acetonitrile solution of n-Bu_4_NPF_6_, as depicted in Figure 1b. The **PDIPN** and **PDIPNSO_3_Na** exhibit onset oxidation potentials at −0.73 eV and −0.79 eV, the HOMO of **PDIPN** and **PDIPNSO_3_Na** are estimated to be −5.59 eV and −5.67 eV, respectively [44,45]. Meanwhile, we can calculate the LUMO of **PDIPN** and **PDIPNSO_3_Na** to be −3.67 eV and −3.61 eV according to their onset reduction potentials; the detailed CV data are provided in Table S1.

**Figure 2:**
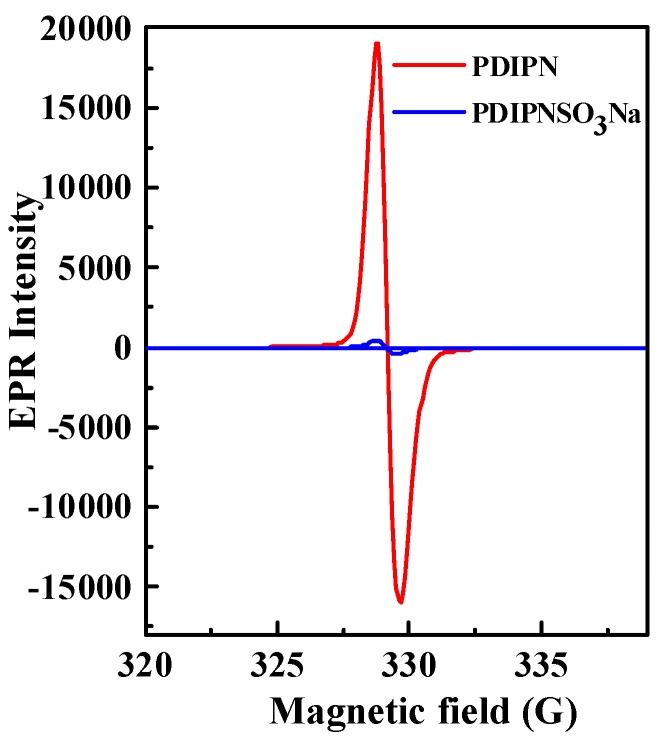
Electron paramagnetic resonance (EPR) spectra of the **PDIPN** and **PDIPNSO_3_Na** film.

To obtain further insight into the n-type self-doping behavior of **PDIPN** and **PDIPNSO_3_Na**, the electron paramagnetic resonance (EPR) spectra are characterized. In Figure 2, obvious narrow line shape signals at about 330G can be detected both in **PDIPN** and **PDIPNSO_3_Na** films, these signals are consistent with the presence of unpaired electrons [43]. Moreover, in comparison to the **PDIPNSO_3_Na**, the EPR signal of **PDIPN** is immensely stronger. These phenomena should because the density of negative charge of **PDIPNSO_3_Na** has been significantly reduced owing to the electron-withdrawing sulfonate of the branched chain, hence inhibiting the electron from transferring to the perylene diimidess nucleus [42]. The result of the EPR is quite in agreement with that of the UV–Vis measurement. Furthermore, we can easily draw a conclusion that both **PDIPN** and **PDIPNSO_3_Na** films possess n-type self-doping effect, and the **PDIPN** film show stronger n-type self-doping compared to that of **PDIPNSO_3_Na**. The schematic diagram of self-doping process is shown in Scheme 1.

**Figure 3:**
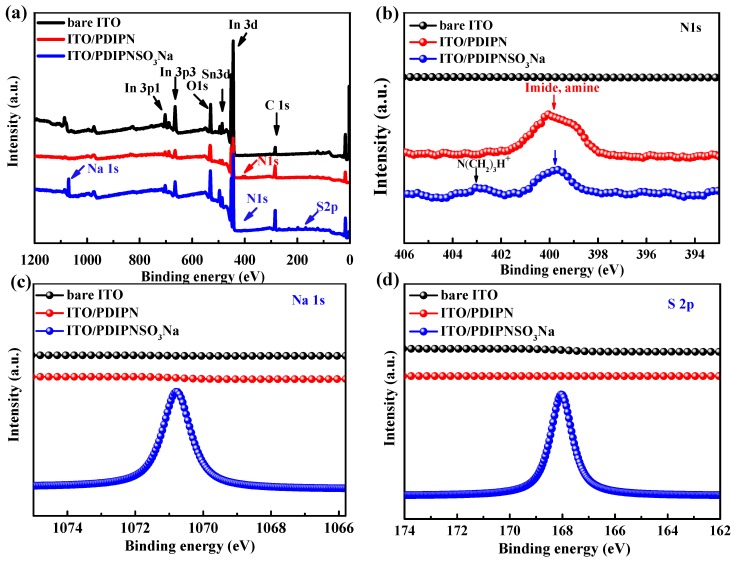
(**a**) Survey X-ray photoelectron spectra of bare ITO, ITO/**PDIPN** and ITO/**PDIPNSO_3_Na** (**b**) N 1s, (**c**) Na 1s, (**d**) S 2p.

The X-ray photoelectron spectra (XPS) of pristine ITO and ITO/ETLs are conducted to investigate the interfacial interaction, as displayed in Figure 3a. Figure 3b is the N 1s XPS graph of the ITO, ITO/**PDIPN** and ITO/**PDIPNSO_3_Na**. Obviously, there is no signal peak detected for bare ITO. Simultaneously, a broad peak at 400.04 eV is detected for ITO/**PDIPN**, which is associated to the N atoms of the imide and amine in **PDIPN**. Besides, for ITO/**PDIPNSO_3_Na**, a broad strong peak at a value of 399.9 eV and a broad weak peak at 403.05 eV are found. Similarly, the peak at 399.9 eV belongs to the imide and amine. However, the new weak peak of 403.05 eV is assigned to N(CH_2_)_3_H^+^. N(CH_2_)_3_ of **PDIPNSO_3_Na** can be partially protonated and form N(CH_2_)_3_H^+^ by reacting with the protons (H^+^) from the water/methanol due to its basicity. From Figure 3c, a strong peak at 1070.80 eV is observed for ITO/**PDIPNSO_3_Na**, which belongs to the sodium sulfonate branched chain. Nevertheless, Na 1s signal peaks cannot be traced in the spectra of ITO and ITO/**PDIPN** due to no Na element. The spectra of S 2p with different ETLs are shown in Figure 3d, there are no peaks in bare ITO and ITO/**PDIPN**, while an obvious peak at 168.04 eV has been found for ITO/**PDIPNSO_3_Na** film, which should be ascribed to the absorption of S atom in -SO_3_Na terminal groups. It indicates that **PDIPN** and **PDIPNSO_3_Na** have been triumphantly synthesized and spin-coated on ITO according to the high-resolution N 1s, Na 1s and S 2p spectra.

**Figure 4:**
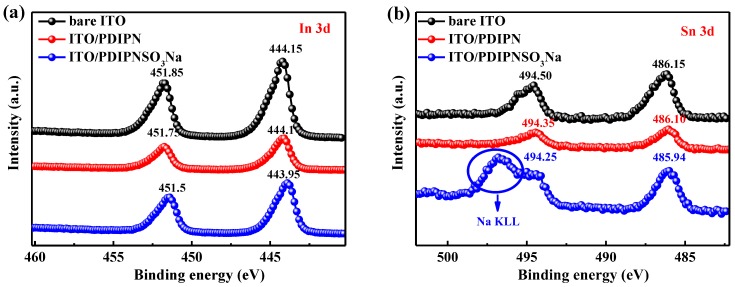
Survey X-ray photoelectron spectra of bare ITO, ITO/**PDIPN** and ITO/**PDIPNSO_3_Na** (**a**) In 3d and (**b**) Sn 3d.

The XPS spectra of the In 3d and Sn 3d are carried out to further characterize the interfacial interactions and interfacial dipole between **PDIPN** and **PDIPNSO_3_Na** and ITO (Figure 4). In comparison to the In 3d of bare ITO (Figure 4a), the resolution peaks of both ITO/**PDIPN** and ITO/**PDIPNSO_3_Na** have moved to lower binding energy. The shift of the In 3d signal indicates that strong interfacial interaction occurs at the interface of the ITO/**PDIPN** and ITO/**PDIPNSO_3_Na**. Similarly, the Sn 3d XPS spectra of ITO, ITO/**PDIPN** and ITO/**PDIPNSO_3_Na** are also studied (Figure 4b). Bare ITO exhibits the Sn 3d peaks at 494.50 eV and 486.15 eV, and the corresponding peaks of ITO/**PDIPN** shift toward lower binding energies to 494.35 eV and 486.10 eV, respectively, and the corresponding peaks of ITO/**PDIPNSO_3_Na** are observed at 494.25 eV and 485.94 eV. Both the In 3d and Sn 3d signals shift to lower binding energy could once again demonstrate that the interfacial dipoles are indeed created and strong interfacial interactions are in the presence of ITO/**PDIPN** and ITO/**PDIPNSO_3_Na** interfaces. Interestingly, a strong broad peak at 497.00 eV has been detected for ITO/**PDIPNSO_3_Na**, which owing to the Na (KLL) Auger peak. The existence of Na (KLL) Auger peak originates from the KLL energy level transition of Na atoms proves that the **PDIPNSO_3_Na** film has been spin-coated on the ITO surface.

**Figure 5:**
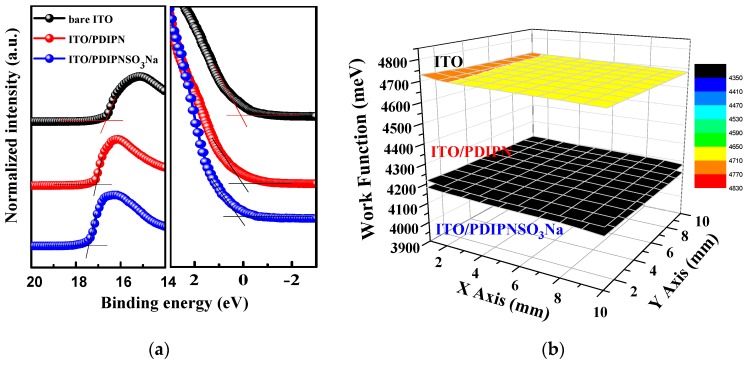
(**a**) Ultraviolet photoelectron spectroscopy (UPS) spectra of the inelastic cutoff region (left) and the HOMO region (right) of bare ITO, ITO/**PDIPN** and ITO/**PDIPNSO_3_Na** ETLs and (**b**) Work function images from Kelvin probe microscopy matrix.

The polar branched chains of the **PDIPN** and **PDIPNSO_3_Na** are inclined to create preferable dipoles at the interface of the metal cathode electrode and active layer, which give rise to the shift of the vacuum-level, thus modifying the work function (WF) of the electrode [46]. The ultraviolet photoelectron spectroscopy (UPS) is carried out to confirm the positive influence of the ETLs **PDIPN** and **PDIPNSO_3_Na** on the WF of ITO (Figure 5a). The high binding energy cutoffs (E_cutoff_) for reference ITO is 16.7 eV. However, the E_cutoff_ of ITO has been shifted toward higher energy to 17.1 eV and 17.4 eV after modified by **PDIPN** and **PDIPNSO_3_Na**, respectively. Simultaneously, there was a slight shift of E_onset._ The E_onset_ values of ITO, ITO/**PDIPN** and ITO/**PDIPNSO_3_Na** are 0.10 eV, 0.05 eV and 0.19 eV. Based on the equation [47].: −HOMO = hν − (E_cutoff_ − E_onset_), hν = 21.22 eV, the HOMO values of ITO, ITO/**PDIPN** and ITO/**PDIPNSO_3_Na** are −4.62 eV, −4.17 eV and −4.01 eV, respectively. Therefore, the WF differences (ΔΦ) among ITO, ITO/**PDIPN** and ITO/**PDIPNSO_3_Na** are −0.45 eV and −0.61 eV. The shifts of the energy levels demonstrate that both the **PDIPN** and the **PDIPNSO_3_Na** can indeed significantly lower the WF of ITO. The decreased WF of ITO originates from the interfacial dipole, manifesting that the interfacial dipole is 0.45 eV for ITO/**PDIPN** and 0.61 eV for ITO/**PDIPNSO_3_Na**. The detailed energy levels data are provided in Table 1. Kelvin probe microscopy (KPM) is used to obtain more information about the impact of the interface dipoles on the WF of ITO, as shown in Figure 5b. Based on the KPM matrix spectra, we can distinctly discover that the WF of ITO has been reduced after modified by **PDIPN** and **PDIPNSO_3_Na**. The bare ITO shows a WF of 4.70 eV. Intriguingly, compared to bare ITO, the WF values of ITO/**PDIPN** and ITO/**PDIPNSO_3_Na** have been decreased by 0.50 eV and 0.54 eV. The KPM results are quite coincide to those of UPS. Moreover, on the basis of the results of UPS and KPM, we can once again verify the interfacial dipole in the interface between ITO/**PDIPN** and ITO/**PDIPNSO_3_Na**. The interfacial dipole is beneficial to reduce the work function of ITO, thus reducing the electron injection barrier and enhancing the efficiencies of charge separation and transfer.

**Figure 6:**
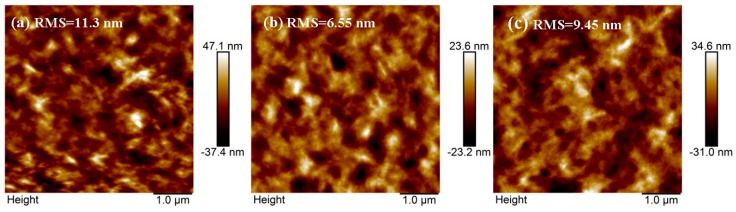
Atomic force microscopy (AFM) tapping mode height images the surface of (**a**) ITO/P3HT:PC_61_BM, (**b**) ITO/**PDIPN**/P3HT:PC_61_BM and (**c**) ITO/**PDIPNSO_3_Na**/P3HT:PC_61_BM.

Surface morphology of the electron transport layer is very important for the charge separation and transfer. The atomic force microscopy (AFM) is measured to characterize the surface morphology of ITO/**PDIPN** and ITO/**PDIPNSO_3_Na**. Figure S3a,b are the height images of ITO/**PDIPN** and ITO/**PDIPNSO_3_Na**, and Figure S3c,d are the corresponding phase images. Delightfully, both **PDIPN** and **PDIPNSO_3_Na** can form homogeneous morphology on the surface of ITO. The root-mean-square (RMS) roughness of ITO/**PDIPN** is 3.36 nm, and the RMS ITO/**PDIPNSO_3_Na** 3.22 nm. Besides, the three-dimensional AFM image of ITO, ITO/**PDIPN** and ITO/**PDIPNSO_3_Na** are presented in Figure S4. The homogeneous and smooth morphology of ETL can facilitate charge transfer and transportation. In addition, the AFM images of the active layer are measured to confirm that whether the surface can become smoother by inserting **PDIPN** and **PDIPNSO_3_Na** than that of without the presence of any ETL (Figure 6). In comparison to ITO/P3HT:PC_61_BM with a RMS roughness of 11.3 nm, the RMS roughness of ITO/**PDIPN**/P3HT:PC_61_BM and ITO/**PDIPNSO_3_Na**/P3HT:PC_61_BM is decreased to 6.55 nm and 9.45 nm, manifesting that both **PDIPN** and **PDIPNSO_3_Na** ETL can exert a positive influence on the morphology of the corresponding active layer. The smoother morphology of the active layer is in favor of charge separation and transfer.

**Figure 7:**
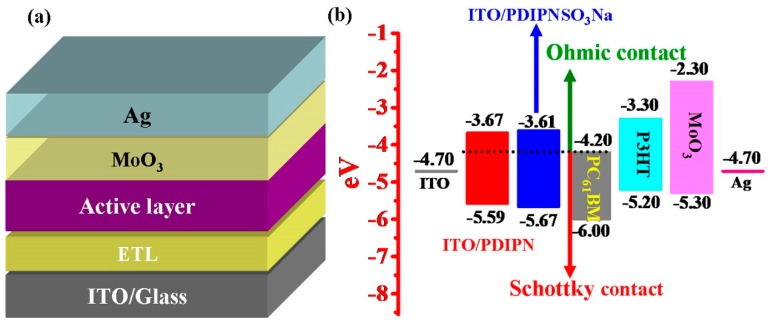
(**a**) The inverted device structure of the PSC. (**b**) Energy-level diagram of the PSCs.

To verify the positive roles of **PDIPN** and **PDIPNSO_3_Na** ETLs in the PSCs, the **PDIPN** and **PDIPNSO_3_Na** ETLs corresponding to inverted devices are fabricated. The schematic representation of the inverted device structure and an energy levels diagram of the PSCs are shown in Figure 7. From the energy levels diagram, we can clearly observe that both the LUMO levels of ITO/**PDIPN** and ITO/**PDIPNSO_3_Na** are higher than that of PC_61_BM, indicating that ohmic contact can be formed after inserting **PDIPN and PDIPNSO_3_Na** ETLs. Meanwhile, compared to the HOMO level of P3HT, the ITO/**PDIPN** and ITO/**PDIPNSO_3_Na** have lower HOMO levels, revealing that both **PDIPN and PDIPNSO_3_Na** ETLs can block the hole transport.

**Figure 8:**
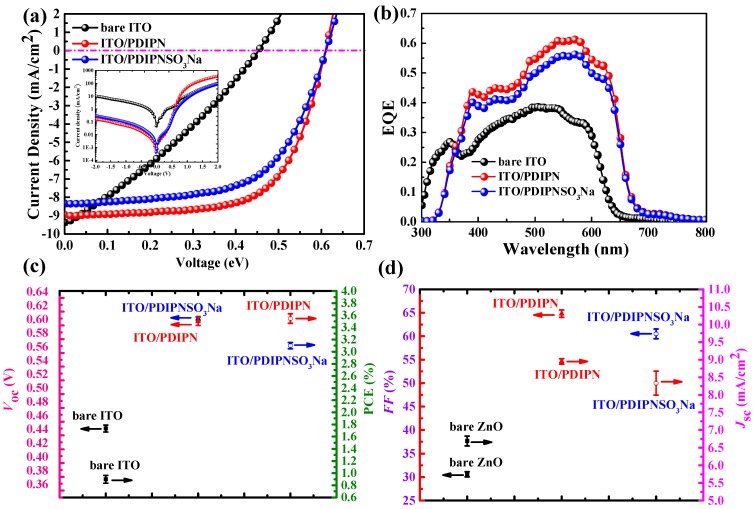
(**a**) *J*−*V* curves of devices based on P3HT:PC_61_BM with various ETLs under the illumination of AM1.5G, 100 mW cm^−2^ (inset: under dark conditions), (**b**) EQE characteristics, (**c**) The standard deviations error bars of *V*_oc_ and PCE and (**d**) the standard deviations error bars of and FF and *J*_sc_.

The *J*-*V* spectra of the device based on ITO, ITO/**PDIPN** and ITO/**PDIPNSO_3_Na** are described in Figure 8a, and the relevant device performance data with different ETLs and different thicknesses are filled in Table S2. The *J*−*V* characteristics of the devices of ITO/**PDIPN** and ITO/**PDIPNSO_3_Na** with different thicknesses are supplemented in Figure S5. The device with ITO obtains a PCE of 0.9%. Delightfully, after modified by **PDIPN** (11 nm), the PCE has been dramatically increased to 3.5% with a *V*_oc_ of 0.61 V, a *J*_sc_ of 8.95 mA/cm^2^ and a fill factor (FF) of 64.8%. Meanwhile, the device with ITO/**PDIPNSO_3_Na** (11 nm) shows a PCE of 3.1%, which is significantly improved compared to that of ITO. It is noteworthy that all the device parameters based on ITO/**PDIPN** and ITO/**PDIPNSO_3_Na**, including *V*_oc_, *J*_sc_, PCE, and FF parameters have been significantly enhanced compared to that of bare ITO. The enhanced device properties should due to the improved interfacial contact and n-type self-doping effect. Manifestly, the photovoltaic performance of ITO/**PDIPN** is slightly higher than that of ITO/**PDIPNSO_3_Na** under the same condition, the higher PCE originates from the higher *J*_sc_ and FF. Compared to ITO/**PDIPNSO_3_Na**, the better performance of ITO/**PDIPN** could owing to its stronger n-type self-doping effect. As we known, the higher n-type self-doping effect is favorable for charge transfer, thus enhancing the *J*_sc_ and FF of the corresponding device. Moreover, when the thicknesses of the ITO/**PDIPN** and ITO/**PDIPNSO_3_Na** are increased to 28 nm, the devices can still achieve an acceptable PCE of 3.1% and 2.8%, respectively. The n-type essence of **PDIPN** and **PDIPNSO_3_Na** can endow them with thickness-insensitive superiority. The thickness-insensitive advantages can make them more suitable for large-scale commercial printing production. To further discuss the effects of the **PDIPN** and **PDIPNSO_3_Na** on the photovoltaic properties, the dark *J*−V curves are studied in the inset of Figure 8a. Encouragingly, the dark current densities of ITO/**PDIPN** and ITO/**PDIPNSO_3_Na** ETLs are dramatically smaller than that of bare ITO under the reverse bias, suggesting that the charge recombination has been significantly inhibited by the contribution of **PDIPN** and **PDIPNSO_3_Na** ETLs. The dark *J*−V curves can further manifest that the preferable interfacial dipoles originate from the polar branched chain and n-type self-doping effect of **PDIPN** and **PDIPNSO_3_Na** can efficiently suppress the leakage current, therefore increasing the charge injection efficiency of the devices. The research results of the dark *J*−V curves are in accordance with that of illuminated *J*−V.

The external quantum efficiency (EQE) has been measured to make further consideration of the interfacial dipoles on the *J*_sc_, as presented in Figure 8b. From the skeleton diagram of the EQE, we can clearly see that both ITO/**PDIPN** and ITO/**PDIPNSO_3_Na** show more outstanding external quantum efficiency between 350 and 700 nm than bare ITO. Notably, ITO/**PDIPN** ETL exhibits the highest value among the three devices. The results from EQE are quite in consistent with those acquired from illuminated and dark *J*−V characteristic. In brief, due to their interfacial modulating and thickness-insensitive properties, both **PDIPN** and **PDIPNSO_3_Na** may become potential candidates as ETLs in polymer solar cells, especially for large-scale solution-processed PSCs. The integrated current values from EQE for ITO/**PDIPN** and ITO/**PDIPNSO_3_Na** are 8.90 mA/cm^2^ and 8.19 mA/cm^2^. The standard deviations error bars of *V*_oc_, PCE*, FF* and *J*_sc_ are shown in Figure 8c,d, and the detailed information are shown in Table 2.

**Figure 9:**
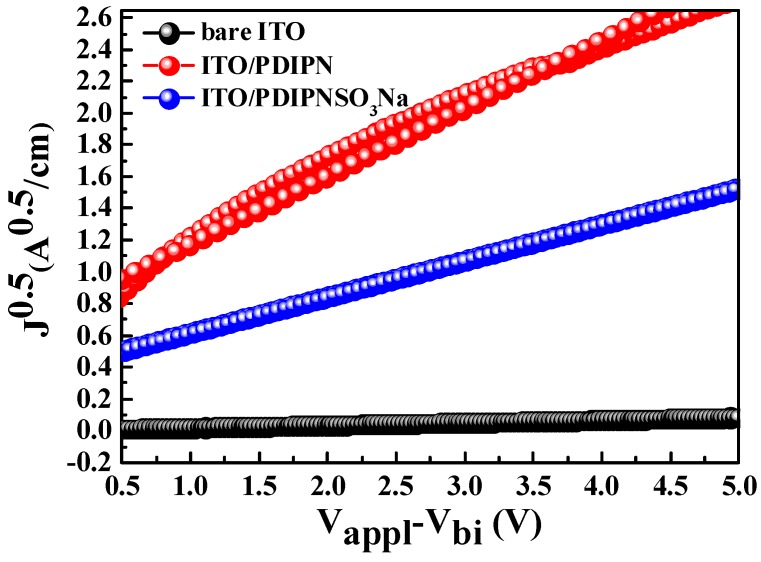
*J*^0.5^−V characteristics of electron-only devices ITO/ETLs/P3HT:PC_61_BM/Al with bare ITO, ITO/**PDIPN** and ITO/**PDIPNSO_3_Na** ETLs.

The *J*−V characteristics of electron-only devices based on P3HT:PC_61_BM blends are measured to get more information regarding electronic mobility, as displayed in Figure 9. The bare-ITO device shows a low electronic mobility of 9.45 × 10^−7^ cm^2^ V^−1^ s^−1^. However, when inserting **PDIPNSO_3_Na** as ETL, the electronic mobility has been dramatically increased to 1.61 × 10^−^^4^ cm^2^ V^−1^ s^−1^. Moreover, in contrast to ITO/**PDIPNSO_3_Na**, the device with ITO/**PDIPN ETL** exhibits better electronic mobility of 4.18 × 10^−^^4^ cm^2^ V^−1^ s^−1^, which should be ascribed to the latter possesses higher n-type self-doping effect, thus supporting higher electronic mobility and higher *J*_sc__._ These results agree well with the electron paramagnetic resonance and *J*–*V* spectra.

## 3. Conclusions

In conclusion, two new n-type water/alcohol-soluble thickness-insensitive ETLs **PDIPN** and **PDIPNSO_3_Na** have been first designed and synthesized as ETL in PSCs. The appropriate energy levels and WF tuning properties of **PDIPN** and **PDIPNSO_3_Na** cause the corresponding PSCs to have acceptable PCE on a relatively broad range of interlayer thickness, especially in the device based on **PDIPN** ETL. Compared to the counterpart **PDIPNSO_3_Na**, **PDIPN** with higher density of an unpaired electron exhibits higher n-type self-doping effect, thus, contributing to higher *J*_sc_, FF and PCE. In comparison to ITO-only devices, the PCE of the device based on ITO/**PDIPN** and ITO/**PDIPNSO_3_Na** ETLs has been increased from 0.9% to 3.1% and 3.5%, respectively. The outstanding properties of **PDIPN** and **PDIPNSO_3_Na** should make them potential candidates as ETL in solution-processed high-efficiency organic photovoltaic devices.

## Figures and Tables

**Table 1:** Energy levels of the ITO, ITO/**PDIPN** and ITO/**PDIPNSO_3_Na**.

ETL	E_cutoff_ (eV)	E_oneset_ (eV)	HOMO (eV)	^a^ ΔΦ (eV)	KPM (eV)
ITO	16.7	0.10	−4.62	-	4.70
ITO/**PDIPN**	17.1	0.05	−4.17	−0.45	4.20
ITO/**PDIPNSO_3_Na**	17.4	0.19	−4.01	−0.61	4.16

^a^ ΔΦ means the difference in the work function of ITO, ITO/**PDIPN** and ITO/**PDIPNSO_3_Na** according to the UPS, −HOMO = hν − (E_cutoff_ − E_onset_), hν = 21.22 eV.

**Table 2:** Device Performance of the Inverted P3HT:PC_61_BM Polymer Solar Cells with Various ETLs.

Cathode Buffer Layer	*V*_oc_ (V)	*J*_sc_ (mA/cm^2^)	*FF* (%)	PCE (%)
ITO	0.45 ± 0.005	6.69± 0.138	30.6 ± 0.54	0.9 ± 0.064
ITO/**PDIPN** (11 nm)	0.61 ± 0.003	8.95 ± 0.078	64.8± 0.79	3.5 ± 0.067
ITO/**PDIPNSO_3_Na** (11 nm)	0.61 ± 0.007	8.33 ± 0.341	60.5 ± 1.03	3.1 ± 0.052

The device parameters of each device are obtained from 10 devices, and the ± refer to the standard deviation.

## Supplementary Materials

Supplementary materials can be found at https://www.mdpi.com/2073-4360/11/4/655/s1.
