# Supplementary material for: Water/Alcohol Soluble Thickness-Insensitive Hyperbranched Perylene Diimide Electron Transport Layer Improving the Efficiency of Organic Solar Cells
Source: Polymers (Basel). 2019 Apr 10;11(4):655. doi: 10.3390/polym11040655 (PMC6523356; doi:10.3390/polym11040655)
Supplement: Supplementary file 1 [file polymers-11-00655-s001.pdf]

Water/Alcohol Thickness-insensitive Hyperbranched Perylene Diimides Electron Transport Layer Improving the Efficiency of Organic Solar Cells

Dan Zhou*[1], Fei Yang[1], Yuancheng Qin[1], Rong Zhong[1], Haitao Xu[2], Yongfen Tong[1], Yubao Zhang[3], Qin Zhang[3], Mingjun Li[1], Yu Xie*[1]

[1]Key Laboratory of Jiangxi Province for Persistent Pollutants, Control and Resources Recycle, Nanchang Hangkong University, 696 Fenghe South Avenue, Nanchang 330063, China

[2]College of Materials Science and Engineering, Nanchang Hangkong University, 696 Fenghe Avenue, Nanchang 330063, China

[3]School of Measuring and Optical Engineering, Nanchang Hangkong University, Nanchang 330063

Corresponding author. E-mail: zhoudan@nchu.edu.cn (D. Zhou), xieyu_121@163.com (Y. Xie)

EXPERIMENTAL SECTION

Materials:

3,4,9,10-Perylenetetracarboxylic diimides, zinc acetate, imidazole, tetraethylene pentamine, NaH, methanol and 1,4-butanesultone were purchased from Alfa, Aldrich or TCI. MoO_3 (99.99%) and Ag (99.99%) were purchased from Alfa Aesar. Poly(3-hexylthiophene) (P3HT, Mw=48300 g mol-1, 2.0-2.4 polydispersity, regiogegular: head-to-tail, 99% purity) and [6,6]-phenyl-C_{60}-butyric acid methyl ester ($PC_{61}BM$, 99.5% purity) were purchased from Alfa or Bayer Inc and used without further purification. Indium-tin oxide (ITO) glass was purchased from Delta Technologies Limited. Tetrahydrofuran (THF) was dried over sodium.

Device Fabrication

The P3HT/$PC_{61}BM$ devices were manufactured with the structure of Glass/ITO/ETL/P3HT:$PC_{61}BM/MoO_3$/Ag. The conductive ITO substrates were sequentially cleaned with ultrasonication in acetone, detergent, deionized water, and

isopropanol. After the ITO substrates were dried by nitrogen purging and treated the surface with UV ozone for 15 min, the **PDIPN** and **PDIPNSO$_3$Na** (1.0 mg mL^{-1}) dissolved in methanol solutions were spin-coated on the ITO at 3000 r/min for 1 min. The active layer P3HT/PC$_{61}$BM (1:0.8 w/w), a blend solution of 10 mg of P3HT and 8 mg of P$_{61}$CBM dissolved in 0.5 mL o-dichlorobenzene solution with a concentration of 20 mg mL^{-1} and was prepared and by spin-casted at 800 rpm for 30 s. And then the film was dried in the glove box for 2 hours, subsequently annealed at 150 °C for 10 min in a nitrogen-atmosphere glove box. Finally, the anode buffer layer MoO$_3$ (7 nm) and Ag (90 nm) electrode was sequentially deposited by thermal evaporation. The effective area of each cell was 0.04 cm^2. The current−voltage (*J*−V) curve was measured by a Keithley 2400 Source Meter under simulated solar light (100 mW/cm^2, AM 1.5 G, Abet Solar Simulator Sun 2000).

Synthesis

The detailed synthetic routes of the target compounds are displayed in **Scheme S1**. The **PDIPN** was obtained by one-step finished reaction between 3,4,9,10-perylenetetracarboxylic diimides and tetraethylene pentamine. Then, **PDIPNSO$_3$Na** was prepared by the substitution reaction between **PDIPN** and 1,4-butanesultone. The chemical structures of the **PDIPN** and **PDIPNSO$_3$Na** are confirmed by ^{1}H nuclear magnetic resonance spectra (^{1}H NMR) (**Figure S1**) and UV-Vis absorption (**Figure 1**). Due to the hydrophilic branched chains encasing the hydrophobic perylene diimides nucleus, the nuclear magnetic resonance signal peaks of the benzene ring of **PDIPN** and **PDIPNSO$_3$Na** can't be detected. However, from the UV-Vis spectra, we can clearly observe strong absorption band at about 400-600 nm, which are attributed to the absorption of perylene diimides nucleus.

Synthesis of PDIPN

Under the protection of nitrogen, 3,4,9,10-perylenetetracarboxylic diimides (2.50 g, 6.37 mmol), catalytic amount of zinc acetate (127 mg), imidazole (10 g) and tetraethylene pentamine (2.89 g, 15.27 mmol) were added in a 250 dried 250 mL round bottom flask, the reaction mixture was stirred at 135 °C overnight under an nitrogen atmosphere. After cooling to room temperature, the reaction solution was

poured into plenty of acetone solution, and refluxed for 3h. The imidazole and excessive tetraethylene pentamine were removed by filtration, and the filter residue was collected. The obtained filter residue was dissolved in methanol and purified by neutral aluminium oxide column. And the solvent was evaporated, dried over vacuum and obtained 3.32 g purple solid (yield: 71%)。 ^{1}H NMR (400 MHz, CD_3OD-d_4), (ppm): 3.645-3.489 (m, 4H), 1.928-1.698 (m, 28H), 1.293-0.997(m,10H).

Synthesis of PDIPNSO$_3$Na.

PDIPN (0.73 g, 1.0 mmol) and dried THF (60 mL) were added to 250 mL round bottom flask under the nitrogen atmosphere, the reaction mixture was cooled to 0 °C in an ice bath, then NaH 1.5 g (60%) was slowly added to the solution and stirred at 0 °C for 0.5 h, the solution was gradually warmed to room temperature and stirred for 6-8h. Then, 1,4-butanesultone (13.6 g, 0.1mol) was added to the reaction mixture, and the reaction solution was reacted at 70 ° C overnight. After cooling to room temperature, the reaction solution was poured into plenty of acetone solution and filtered, the obtained filter residue was washed with acetone and dried under vacuum yield purple red solid (71%).

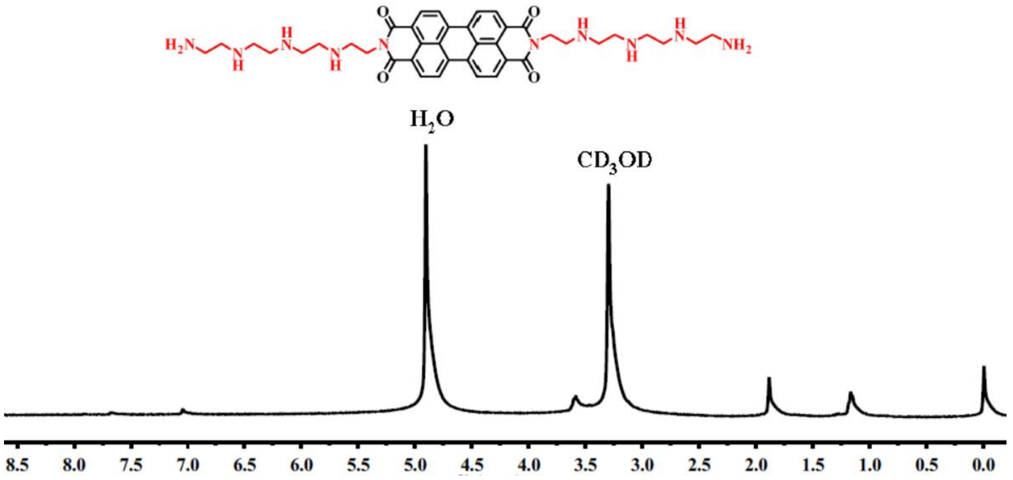

Figure S1. The ^{1}H NMR spectrum of the **PDIPN** recorded in deuterated methanol solution.

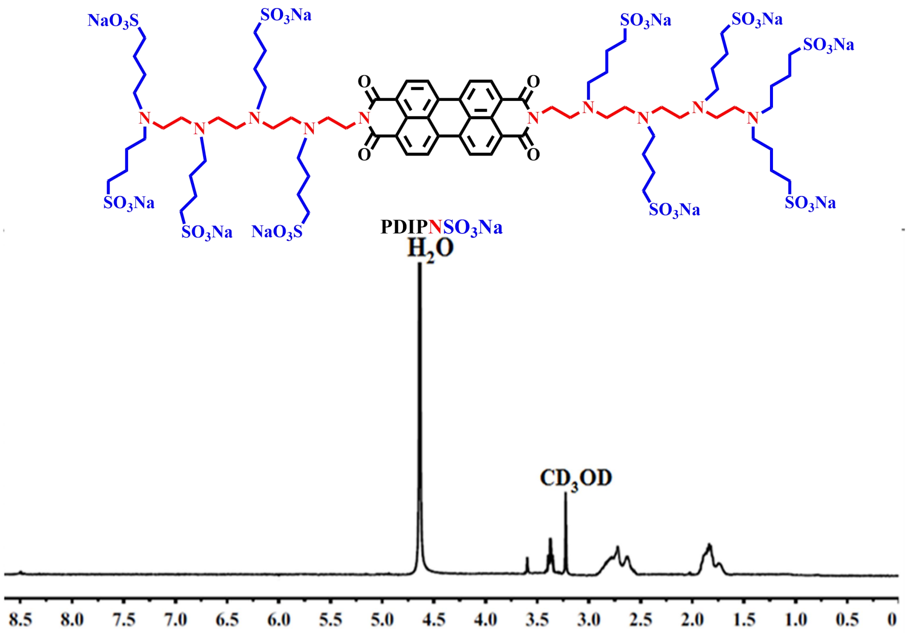

Figure S2. The ^{1}H NMR spectrum of the **PDIPNSO₃Na** recorded in deuterated methanol solution.

Table S1. Energy levels of **PDIPN** and **$PDIPNSO_3Na$** films.

ETL	$E^{onset\ (red)}$(V)	$E^{onset\ (ox)}$(V)	HOMO(eV)[a]	LUMO(eV)[a]	E_g^{opt}	E_g^{ec}(eV)[b]
**PDIPN**	-0.73	1.19	-5.59	-3.67	2.02	1.92
**$PDIPNSO_3Na$**	-0.79	1.27	-5.67	-3.61	2.00	2.06

[a]HOMO and LUMO energy values are calculated from the onset of the corresponding redox waves, $E_{LUMO/HOMO} = -e(E^{onset\ (red/ox)} + 4.4)$ eV. [b]E_g^{ec} represents the energy levels differences between LUMO and HOMO from cyclic voltammetry.

Table S2. Photovoltaic Performance of Traditional P3HT:$PC_{61}BM$ Solar Cells with bare ITO, ITO/**PDIPN** and ITO/**PDIPNSO$_3$Na** ETLs.

Active Layer	ETLs (thickness)	V_{oc} (V)	J_{sc} (mA/cm^2)	FF (%)	PCE (%)
P3HT:$PC_{61}BM$	ITO	0.45	6.690	30.6	0.9
P3HT:$PC_{61}BM$	ITO/**PDIPN** (5 nm)	0.61	8.680	64.9	3.4
P3HT:$PC_{61}BM$	ITO/**PDIPN** (11 nm)	0.61	8.950	64.8	3.5
P3HT:$PC_{61}BM$	ITO/**PDIPN** (28 nm)	0.62	8.550	58.4	3.1
P3HT:$PC_{61}BM$	ITO/**PDIPNSO$_3$Na** (5 nm)	0.58	8.380	60.5	3.0
P3HT:$PC_{61}BM$	ITO/**PDIPNSO$_3$Na** (11 nm)	0.61	8.330	60.5	3.1
P3HT:$PC_{61}BM$	ITO/**PDIPNSO$_3$Na** (28 nm)	0.59	8.550	54.7	2.8

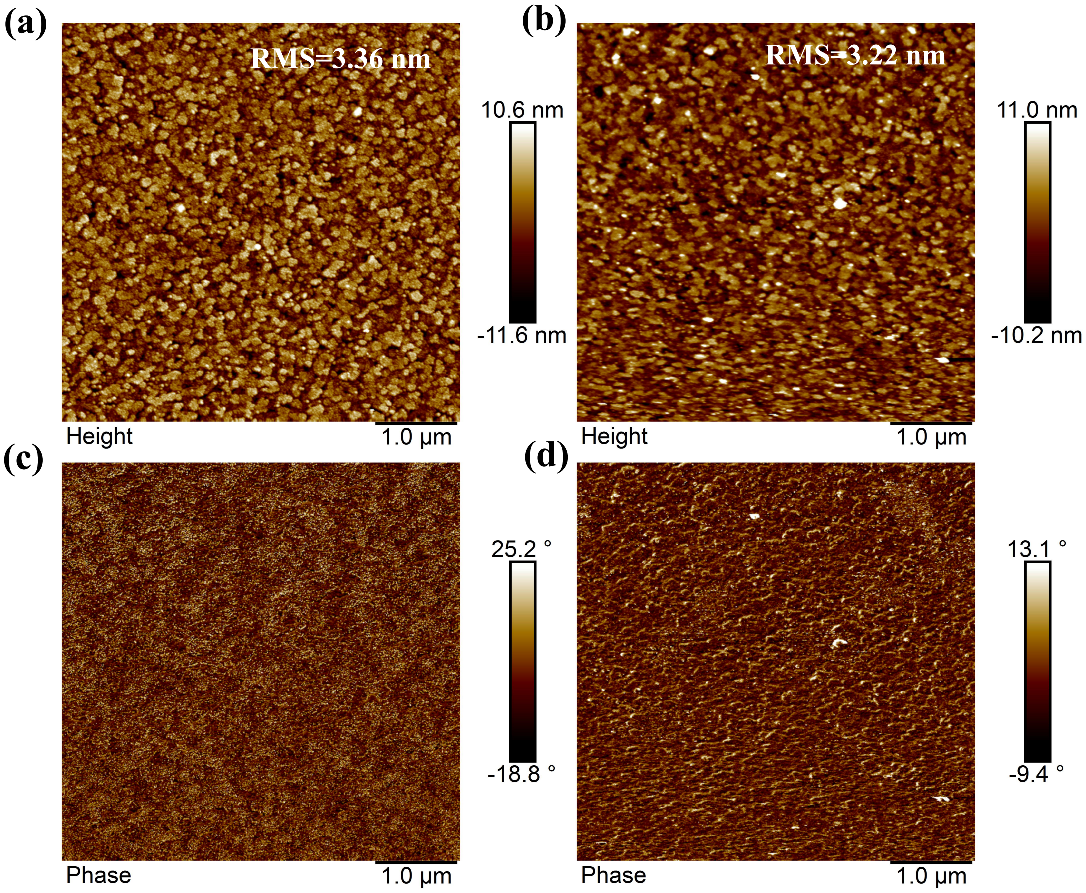

Figure S3. Atomic force microscopy (AFM) tapping mode height images (above) and phase images (below) of the surface of (a, c) ITO/**PDIPN** and (b, d) ITO/**PDIPNSO$_3$Na**.

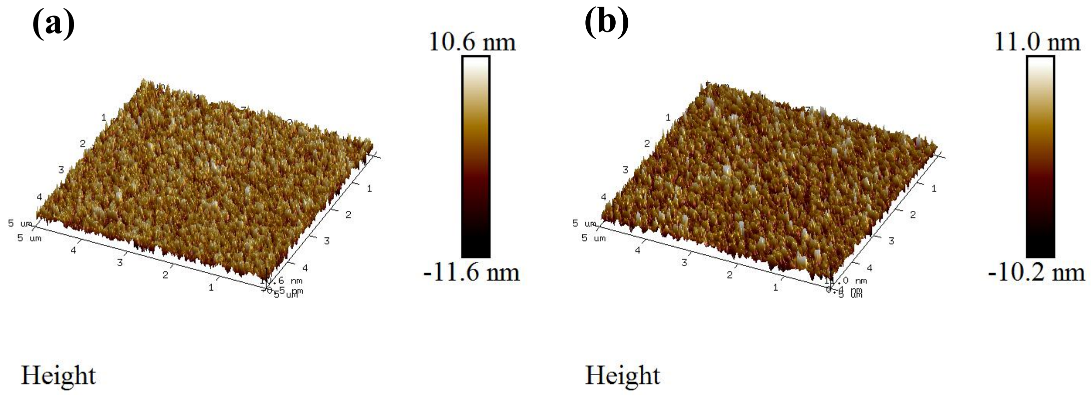

Figure S4. Atomic force microscopy tapping mode three-dimensional image of the surface of (a) ITO/**PDIPN** and (b) ITO/**PDIPNSO$_3$Na** films.

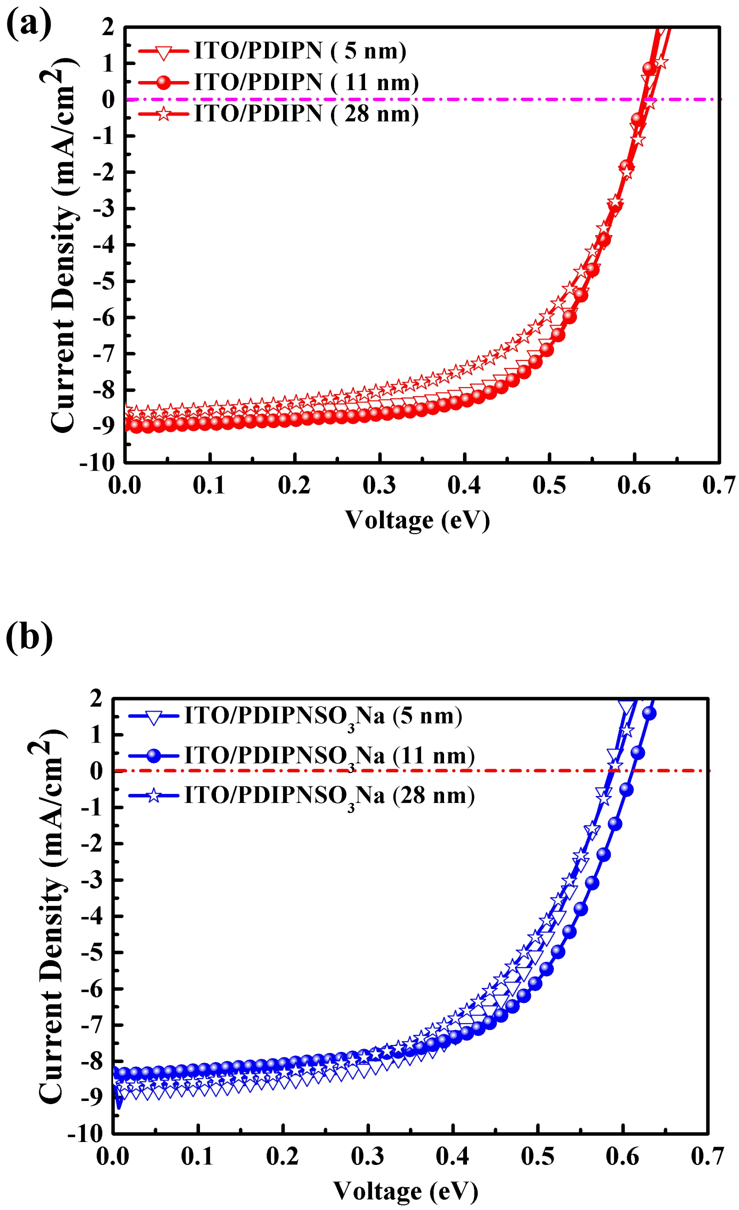

Figure S5. *J*–*V* characteristics of the devices with (a) **PDIPN** and (b) **PDIPNSO$_3$Na** electron transport layers of various thicknesses.
